# Regional CSF volume quantification using deep learning for comparative analysis of brain atrophy in frontotemporal dementia subtypes

**DOI:** 10.3389/fnagi.2025.1631640

**Published:** 2025-09-22

**Authors:** Kyoung Yoon Lim, Soyeon Yoon, Seongbeom Park, Seongmi Kim, Kyoungmin Kim, Jehyun Ahn, Jun Pyo Kim, Hee Jin Kim, Duk L. Na, Sang Won Seo, Kichang Kwak

**Affiliations:** ^1^BeauBrain Healthcare, Inc., Seoul, Republic of Korea; ^2^Alzheimer’s Disease Convergence Research Center, Samsung Medical Center, Seoul, Republic of Korea; ^3^Department of Neurology, Samsung Medical Center, Sungkyunkwan University School of Medicine, Seoul, Republic of Korea; ^4^Department of Health Sciences and Technology, SAIHST, Sungkyunkwan University, Seoul, Republic of Korea; ^5^Department of Digital Health, SAIHST, Sungkyunkwan University, Seoul, Republic of Korea

**Keywords:** frontotemporal dementia, deep learning, cerebrospinal fluid, brain atrophy, magnetic resonance imaging

## Abstract

**Introduction:**

Frontotemporal dementia (FTD) encompasses heterogeneous clinical syndromes, and distinguishing its subtypes using imaging remains challenging.

**Methods:**

We developed a deep learning model to quantify brain atrophy by measuring cerebrospinal fluid (CSF) volumes in key regions of interest (RoIs) on standard MRI scans. In a retrospective study, we analyzed 3D T1-weighted MRI data from 1,854 individuals, including cognitively unimpaired (CU) controls, patients with dementia of the Alzheimer type (DAT), and FTD subtypes: behavioral variant FTD (bvFTD), nonfluent variant primary progressive aphasia (nfvPPA), and semantic variant PPA (svPPA). The model quantified CSF volumes in 14 clinically relevant RoIs and generated age- and sex-adjusted W-scores to express regional atrophy.

**Results:**

Each FTD subtype exhibited a distinct, lateralized atrophy pattern: bvFTD showed widespread bilateral frontal and right-predominant parietal and temporal atrophy; nfvPPA showed left-predominant frontal and parietal atrophy; and svPPA exhibited marked left-lateralized temporal and hippocampal atrophy. All FTD subtypes demonstrated significantly greater CSF expansion in these characteristic regions compared to DAT and CU.

**Discussion:**

This deep learning approach provides a simple, interpretable measure of brain atrophy that differentiates FTD subtypes, requiring only standard MRI with minimal preprocessing, and offers clinical utility.

## Introduction

Neurodegenerative disease is a common pathological hallmark underlying various types of dementia, including Alzheimer’s disease (AD) and frontotemporal dementia (FTD) (Rosen et al., 2002; Whitwell et al., 2007; Erkkinen et al., 2018). Brain atrophy is a key feature of these diseases, characterized by progressive neuronal loss, cortical thinning, and sulcal widening (Im et al., 2008). These structural changes are closely associated with cognitive and behavioral decline, making the quantification of brain atrophy crucial for early diagnosis, progression monitoring, and therapeutic evaluation (Brambati et al., 2009; Marino et al., 2019).

Frontotemporal dementia (FTD) comprises several clinical subtypes, including behavioral variant FTD (bvFTD), semantic variant primary progressive aphasia (svPPA), and non-fluent variant primary progressive aphasia (nfvPPA), each with distinct neuroanatomical atrophy patterns that differ markedly from those observed in AD (Risacher and Saykin, 2013; Ghosh and Lippa, 2015; Erkkinen et al., 2018; Yu et al., 2021; Eldaief et al., 2023; Taylor et al., 2025). While AD is typically associated with atrophy in the hippocampus and medial temporal regions (Ferreira et al., 2017), FTD more often presents with frontal and anterior temporal lobe degeneration, with variability in regional involvement depending on the clinical subtype (Lu et al., 2013; Planche et al., 2023). Identifying and characterizing these subtype-specific patterns of atrophy is critical for improving the differential diagnosis between AD and FTD.

Traditional methods for assessing brain atrophy, such as visual atrophy scales by clinicians, rely on subjective ratings of atrophy based on visual inspection of structural MRI scans (Scheltens et al., 1995; Kipps et al., 2007). While these methods remain prevalent in clinical practice, they are limited by their labor-intensive, time-consuming, susceptibility to inter-rater variability, and lack sensitivity to subtle anatomical changes (Duara et al., 2010; Cavallin et al., 2012; Choe et al., 2024). To overcome these limitations, advanced techniques, such as the measurement of cortical thickness (Fischl and Dale, 2000; Chu et al., 2024) and voxel-based morphometry (VBM) (Ashburner and Friston, 2000; Rosen et al., 2002) have been introduced, offering automated and more quantitative assessments of atrophy. However, these methods often struggle with segmentation inaccuracy due to the subtle intensity differences between gray-white matter boundary, leading to inconsistencies across imaging protocols and scanners (Leung et al., 2010; Pagnozzi et al., 2019), and they typically require complex preprocessing pipelines and spatial normalization steps that can impede their clinical utility due to high computational demands (Tustison et al., 2019; Bhagwat et al., 2021; Zhou et al., 2022).

To address these challenges, cerebrospinal fluid (CSF) volumetry offers a practical alternative: its strong intensity contrast with surrounding tissues enables robust segmentation across sites, and focusing on sulcal and ventricular CSF provides a reliable proxy for cortical atrophy (Kochunov et al., 2008; Im et al., 2008; De Vis et al., 2016). Building on this rationale, we employed a previously proposed deep learning–based segmentation model (Lim et al., 2025) to automatically quantify CSF volumes in 3D T1-weighted MRI scans. This approach serves as a proxy for sulcal widening and ventricular enlargement—key markers of neural tissue loss—while providing a practical and scalable solution aligned with routine clinical workflows. It facilitates intuitive interpretation, rapid clinical integration, and consistent application across diverse imaging settings.

In this study, regional CSF volume measures derived from the model were used to evaluate differences in brain atrophy patterns across three clinical subtypes of frontotemporal dementia (FTD), cognitively unimpaired (CU) individuals, and patients with Alzheimer’s disease (DAT). We hypothesized that each FTD subtype would exhibit distinct CSF volume-based regional atrophy patterns that could be statistically distinguished from those of DAT and CU individuals.

## Materials and methods

### Participants

**Figure 1:**
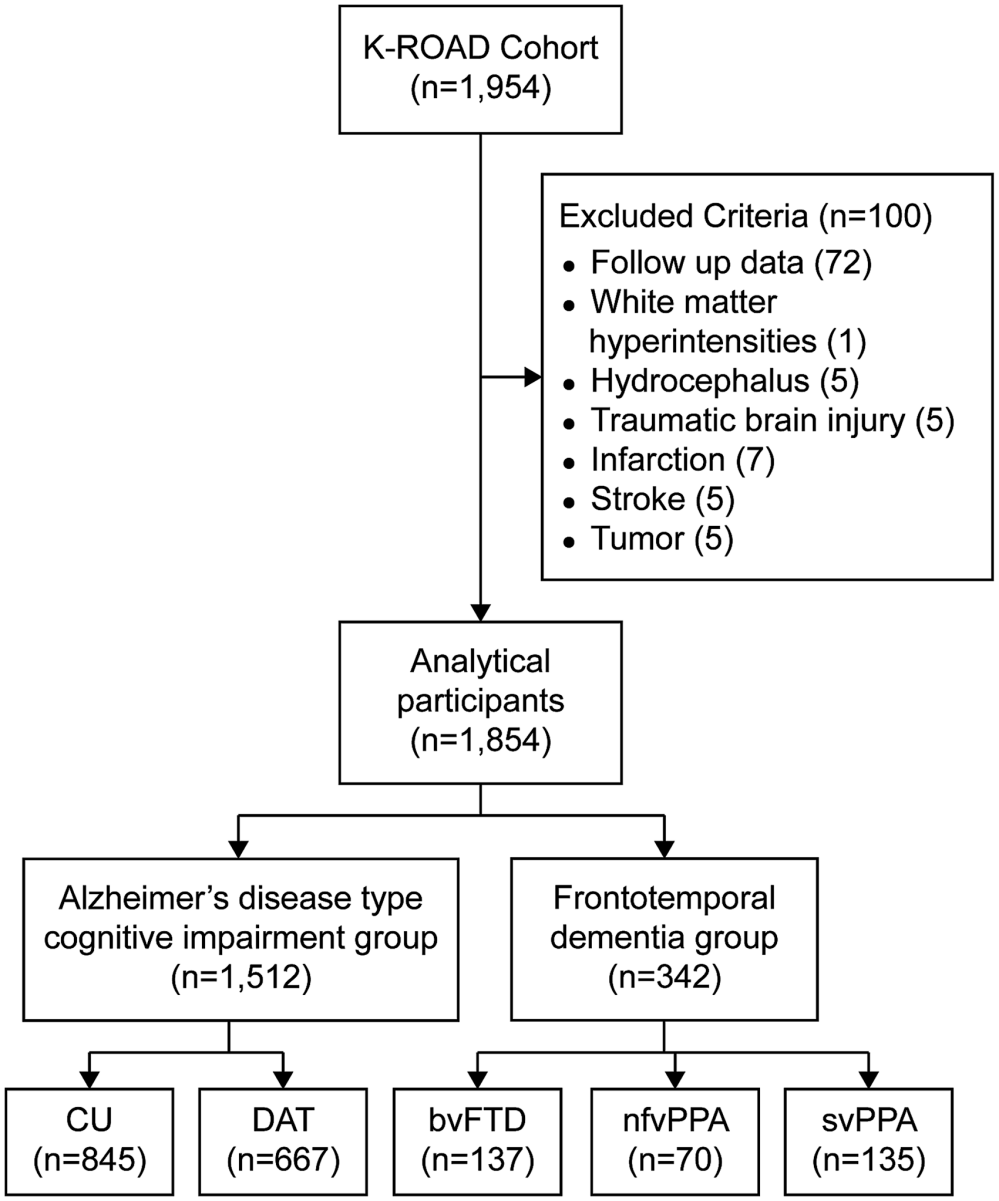
Flowchart of participant inclusion and exclusion. A total of 1,954 participants underwent brain MRI as part of the K-ROAD study between 2016 and 2023. After excluding 100 participants with follow-up scans or structural brain lesions, 1,854 participants were included for analysis. Participants were categorized into the Alzheimer’s disease cognitive impairment group (CU, *n* = 845; DAT, *n* = 667) and the frontotemporal dementia group (bvFTD, *n* = 137; nfvPPA, *n* = 70; svPPA, *n* = 135). CU, cognitively unimpaired; DAT, dementia of Alzheimer’s type; bvFTD, behavioral variant frontotemporal dementia; nfvPPA, nonfluent variant primary progressive aphasia; svPPA, semantic variant primary progressive aphasia.

A total of 1,954 MRI scans for this study were retrospectively collected between 2016 and 2023 from the Korea-Registries to Overcome dementia and Accelerate Dementia Research (K-ROAD) project (Jang et al., 2024). The K-ROAD project was conducted between 2016 and 2023 in collaboration with 25 university-affiliated hospitals across South Korea. Its goal was to establish a genotype–phenotype cohort to advance the development of innovative diagnostic and therapeutic approaches for neurodegenerative diseases, particularly Alzheimer’s disease and related dementias. The participants in the FTD subtypes included those clinically diagnosed with bvFTD, nfvPPA, or svPPA. bvFTD was defined according to established diagnostic criteria (Rascovsky et al., 2011), while nfvPPA and svPPA were diagnosed based on diagnostic criteria for primary progressive aphasia (Gorno-Tempini et al., 2011). All FTD diagnoses were made through a comprehensive evaluation that included clinical course, neurological examination, neuropsychological testing, and brain imaging. CU individuals exhibited no subjective cognitive complaints or functional impairments, with cognitive performance confirmed to be within normal limits through detailed neuropsychological assessments. The diagnosis of DAT was established based on the NIA-AA criteria (McKhann et al., 2011), requiring evidence of substantial cognitive decline, including memory impairment, that compromised independent daily functioning and aligned with Alzheimer’s disease etiology. Of these, 72 participants with follow-up scans and 28 participants with structural brain lesions were excluded from the analysis (Figure 1). Structural lesions were defined based on the presence of any of the following: (1) white matter hyperintensities due to radiation injury, (2) hydrocephalus; (3) traumatic brain injury; (4) territorial infarction; (5) stroke; and (6) brain tumor.

The study protocol received approval from the Institutional Review Board (IRB) of SMC (IRB No. 2021–02-135). Written informed consent was obtained from each participant, and all procedures were conducted in accordance with the approved guidelines.

### Acquisition of 3D T1 images

All participants underwent brain MRI at each participating center using a standardized imaging protocol for three-dimensional (3D) T1-weighted turbo field echo sequences acquired on 3.0 T MRI scanners. All images were subsequently centralized and processed at Samsung Medical Center.

### Deep learning-based segmentation method for CSF regions

For the automated segmentation of cerebrospinal fluid (CSF) regions, we employed the 2D nnU-Net framework with a PlainConvUNet architecture (Isensee et al., 2021). The network follows a self-configuring six-stage encoder–decoder U-Net design, in which the encoder progressively reduced spatial resolution while increasing feature dimensionality, and the decoder reconstructed spatial details through transposed convolutions. Multi-scale feature fusion is achieved through skip connections between corresponding encoder and decoder levels. Each stage consists of two plain convolutional layers with a kernel size of 3 × 3, followed by instance normalization and a leaky rectified linear unit. The nnU-Net framework automatically configures preprocessing, data augmentation, training schedules, and postprocessing pipelines based on the dataset’s properties, ensuring robust and reproducible segmentation performance without extensive manual tuning.

MR images used for model training and testing were obtained from multiple sources, including SMC; the Alzheimer’s Disease Neuroimaging Initiative (Petersen et al., 2010); the International Consortium for Brain Mapping (Mazziotta et al., 2001); the Information eXtraction from Images project; and the Open Access Series of Imaging Studies (LaMontagne et al., 2019) (see Supplementary Table 1 for details). All scans were converted to 3D NIfTI format and processed using the SynthSeg in FreeSurfer (version 7.4.2) (Billot et al., 2023), which simultaneously generated silver-standard segmentation masks and resampled the images to isotropic 1mm^3^ × 1mm^3^ × 1 mm^3^ voxel spacing, resulting in standardized 3D volumes for supervised training. The silver standard masks defined the following regions as RoIs: CSF adjacent to the cortical gray matter in the bilateral frontal (L_Frontal, R_Frontal), occipital (L_Occipital, R_Occipital), parietal (L_Parietal, R_Parietal), and temporal (L_Temporal, R_Temporal) lobes, as well as ventricular spaces such as the bilateral anterior lateral ventricles (L_Anterior_LV, R_Anterior_LV), posterior lateral ventricles (L_Posterior_LV, R_Posterior_LV), and the CSF surrounding the left and right hippocampal regions (L_Hippocampal, R_Hippocampal).

Segmentation was performed using 5-fold cross-validation. Model training was performed over 200 epochs with a batch size of 64, using stochastic gradient descent as the optimizer, a composite loss function combining Dice and cross-entropy losses, a learning rate of 1 × 10^−2^, and weight decay of 3 × 10^−5^. Model performance was assessed using the Dice similarity coefficient (DSC), a measure of spatial overlap between the prediction and the silver-standard mask, as follows:


$$\mathrm{DSC}=\frac{2\times \mathrm{TP}}{2\times \mathrm{TP}+\mathrm{FP}+\mathrm{FN}}$$

where TP, FP, and FN represent true positives, false positives, and false negatives, respectively.

### Quantification of brain atrophy based on regional CSF volume

To assess brain atrophy, cerebrospinal fluid (CSF) volumes were obtained from 14 predefined regions of interest (RoIs) segmented on each participant’s raw T1-weighted MRI scans using our automated processing pipeline, which includes the segmentation model, as detailed in Supplementary Figure 1. For each participant, the CSF volume of each RoI (denoted as RoI_CSF_Vol) was normalized by intracranial volume (ICV) to account for individual variability in head size. To further normalize these values across participants, normalized RoI_CSF_Vol measures were converted to W-scores using multiple linear regression models that included age and sex as covariates (La Joie et al., 2012). The W-score is calculated as follows:


$$W-\text{scor}e_{\mathrm{RoI}}=-\frac{V_{\mathrm{RoI}}-E_{W\_\mathrm{RoI}}(A,S)}{\sigma_{W\_\mathrm{RoI}}}$$

where $E_{W\_\mathrm{ROI}}(A,S)$ is expected CSF_Vol for a given RoI in W-score model with age (A) and sex (S), $V_{\mathrm{ROI}}\;$is the participant’s CSF_Vol of RoI derived from segmentation model, and $\sigma_{W\_\mathrm{ROI}}$ is the standard deviation of the residuals in W-score model. Given that increased CSF volume is indicative of brain atrophy, the W-score was inverted to align with this biological interpretation (Lim, Park et al., 2025). Finally, the spatial distribution of brain atrophy was visualized by projecting average W-score maps of each FTD subtype onto a cortical surface.

### Statistical analysis

To compare participant characteristics across group, Chi-square tests were applied to categorical variables, while one-way analysis of variance (ANOVA) was used for continuous variables, including age, years of education, Mini-Mental State Examination (MMSE) scores, and ICV. When significant group differences were found, Bonferroni-adjusted *post hoc* analyses were conducted separately to compare each FTD subtype with CU individuals and patients with DAT. For regional brain atrophy analysis, W-score distributions for each RoI were visualized using boxplots. Group differences in RoI W-scores were assessed using one-way ANOVA, followed by Bonferroni-corrected post hoc comparisons between each FTD subtype and both CU and DAT groups. To quantify the magnitude of group-level differences, Cohen’s *d* effect sizes were calculated for each RoI. All statistical analyses were performed using R software, version 4.4.2.^1^

## Results

### Participant characteristics

The demographic characteristics of participants are presented in Table 1. The cohort consisted of 1,854 participants: 845 CU, 667 DAT, and 342 FTD subtypes (137 bvFTD, 70 nfvPPA, and 135 svPPA). The mean age of the entire cohort was 71.1 ± 8.3 years, with females comprising 58.3%. The mean MMSE score was 23.7 ± 6.2. The average years of education was 11.6 ± 4.7, and the mean intracranial volume (ICV) was 1,481.4 ± 131.4 mL.

**Table 1:** Characteristics of participants.

Characteristics	CU (*N* = 845)	bvFTD (*N* = 137)	nfvPPA (*N* = 70)	svPPA (*N* = 135)	DAT (*N* = 667)
Age, mean ± SD, years^1^	70.7 ± 7.2	66.6 ± 10.7^*†^	68.8 ± 8.6^†^	67.0 ± 8.6^*†^	67.0 ± 8.6
Sex, female, N (%)^2^	516 (61.1%)	58 (42.3%)	37 (52.9%)	66 (48.9%)	403 (60.4%)
Years of education, mean ± SD^1^	12.1 ± 4.5 (*N* = 845)	11.4 ± 4.5 (*N* = 134‡)	11.5 ± 4.3 (*N* = 66‡)	11.4 ± 4.4 (*N* = 131‡)	11.1 ± 5.0
MMSE (Kim et al., 2024), mean±SD^1^	28.3 ± 1.6 (*N* = 845)	20.5 ± 6.1^*^ (*N* = 108‡)	20.6 ± 7.0^*^ (*N* = 53‡)	19.1 ± 8.8^*^ (*N* = 113‡)	19.5 ± 5.1
ICV, mean ± SD, mL^1^	1,486.7 ± 131.3	1,505.0 ± 139.9^†^	1,500.6 ± 108.0	1,482.8 ± 129.1	1,467.0 ± 131.3

Values are expressed as mean ± standard deviation (SD) or number (%) as appropriate. Statistical comparisons for continuous variables^1^ were performed using analysis of variance (ANOVA) followed by Bonferroni post hoc tests. Categorical variables^2^ were compared using Pearson’s Chi-squared test. A p-value < 0.05 was considered significant. ‡ Sample sizes for Years of education and MMSE variables differ from the No. of participants due to missing data: Years of education: bvFTD (*n* = 134), nfvPPA (*n* = 66), svPPA (*n* = 131). MMSE: bvFTD (*n* = 108), nfvPPA (*n* = 53), svPPA (*n* = 113). Summary statistics for the overall cohort are provided in the text. Summary statistics for the overall cohort are provided in the text. CU, cognitively unimpaired; bvFTD, behavioral variant frontotemporal dementia; nfvPPA, nonfluent variant primary progressive aphasia; svPPA, semantic variant primary progressive aphasia; DAT, dementia of Alzheimer’s type; N, number of subjects; SD, Standard deviation; MMSE, Mini–Mental State Examination; ICV, intracranial volume, Asterisk (*), significant difference with cognitively unimpaired individuals; Dagger (†), significant difference with dementia of Alzheimer’s type patients.

### Regional variations in W-scores among diagnostic groups

**Figure 2:**
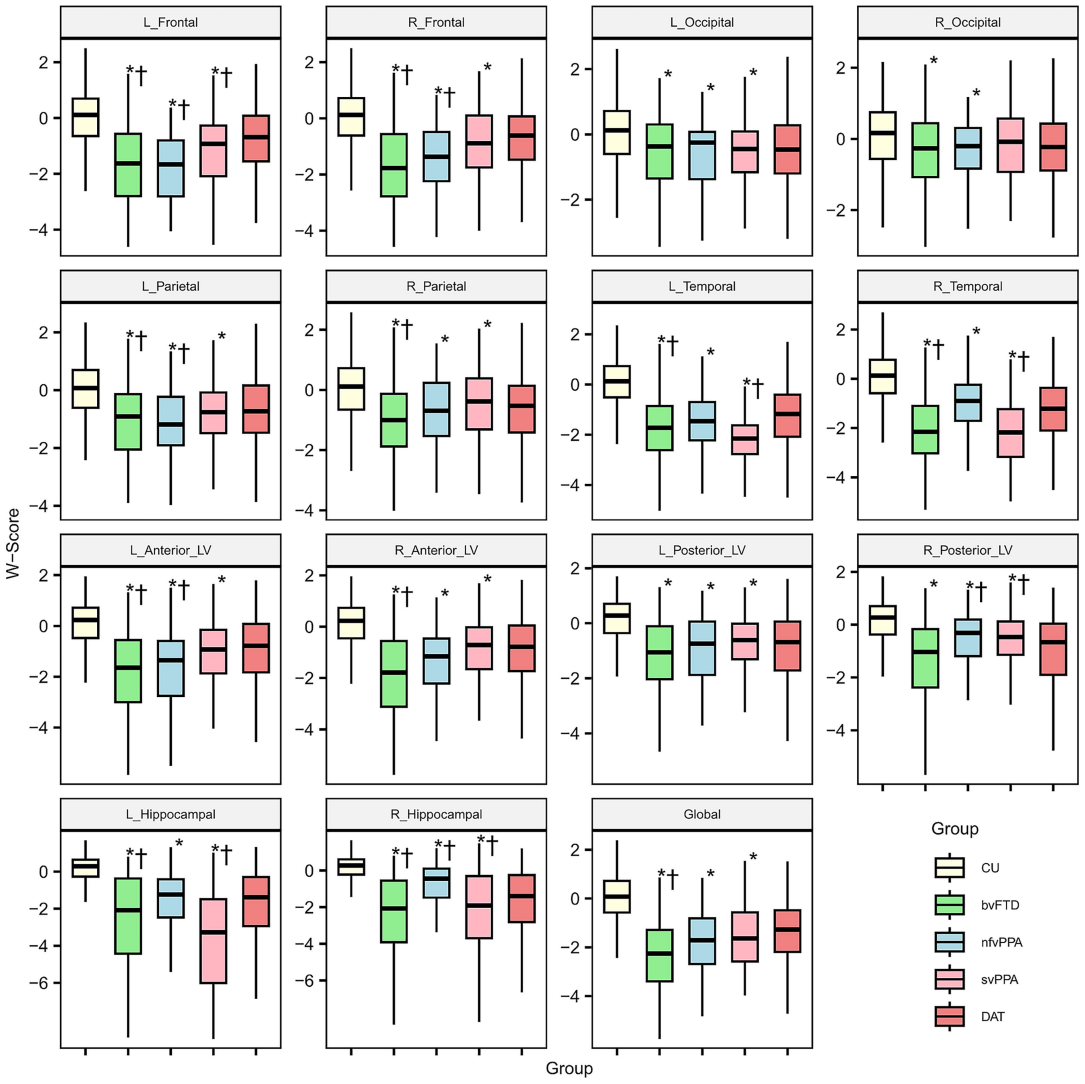
Boxplots of W-scores for cerebrospinal fluid volume of each region of interest. An asterisk (*) indicates significant difference with CU individuals and dagger (†) indicates significant difference with DAT patients. For visual convenience, W-scores beyond ±3 standard deviations were excluded from the boxplots, without affecting the underlying statistical analysis. CU, cognitively unimpaired; bvFTD, behavioral variant frontotemporal dementia; nfvPPA, nonfluent variant primary progressive aphasia; svPPA, semantic variant primary progressive aphasia; DAT, dementia of Alzheimer’s type; CSF, cerebrospinal fluid; L_Anterior_LV, left anterior lateral ventricle; R_Anterior_LV, right anterior lateral ventricle; L_Posterior_LV, left posterior lateral ventricle; R_Posterior_LV, right posterior lateral ventricle; L_Hippocampal, left hippocampal; R_Hippocampal, right hippocampal; L_Frontal, left frontal; R_Frontal, right frontal; L_Temporal, left temporal; R_Temporal, right temporal; L_Parietal, left parietal; R_Parietal, right parietal; L_Occipital, left occipital; R_Occipital, right occipital; Global, sum of all cerebrospinal fluid regions of interest.

The boxplots in Figure 2 illustrate the distribution of W-scores across RoIs for each diagnostic group. CU individuals generally exhibited W-scores indicative of minimal atrophy, while all FTD subtypes demonstrated significantly lower W-scores across all RoIs, except for the right occipital region. Comparisons between DAT and FTD subtypes revealed distinct, region-specific patterns. Specifically, bvFTD showed marked atrophy in the bilateral frontal, parietal, and temporal lobes, as well as in the anterior LVs and hippocampal regions. The patients with nfvPPA demonstrated predominant atrophy in the bilateral frontal, left parietal lobes, and left anterior LV. In contrast, svPPA was characterized by significant degeneration in the left frontal, bilateral temporal lobes, and hippocampal regions.

### Effect sizes and spatial distributions of regional atrophy patterns

**Figure 3:**
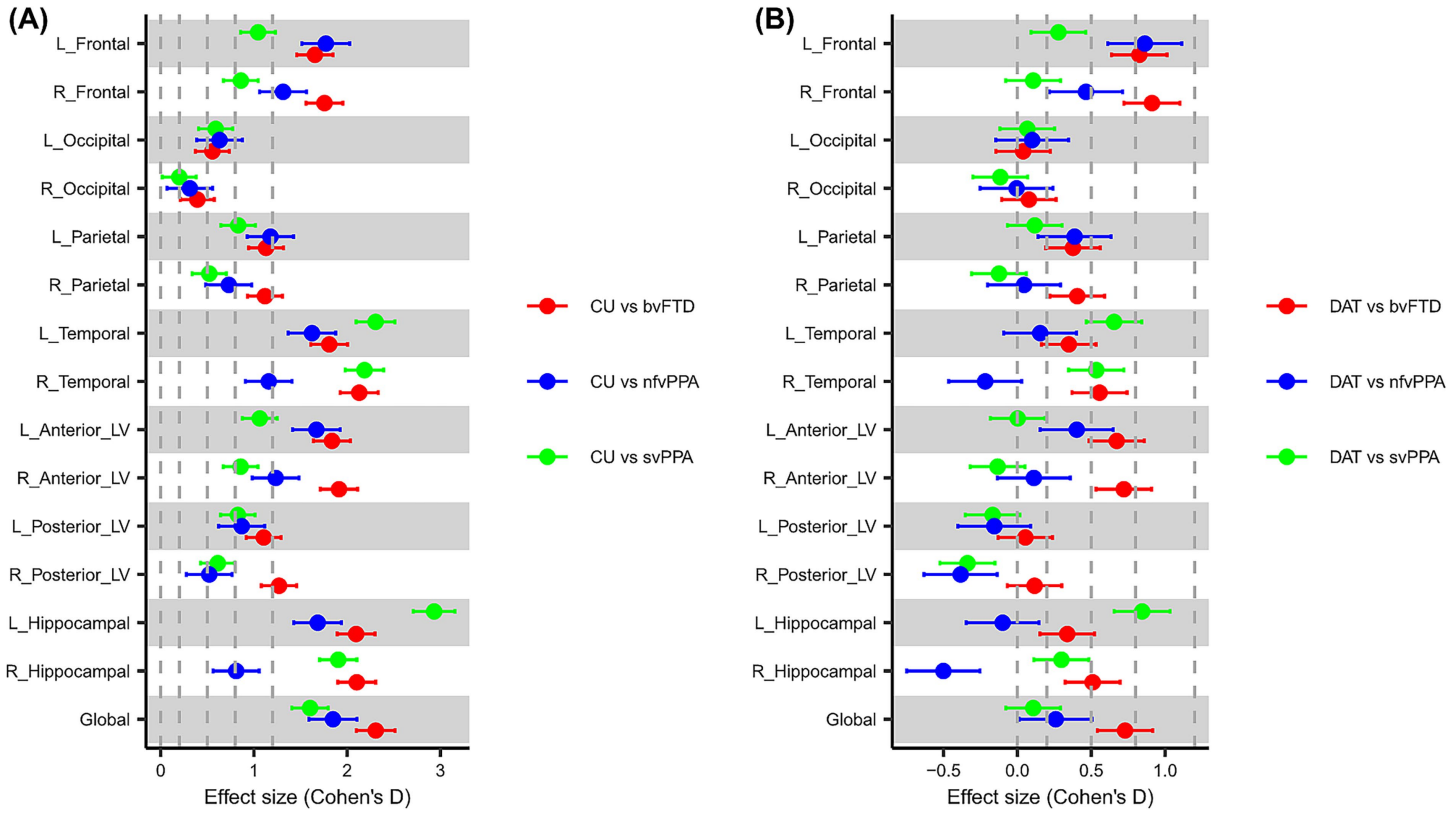
Forest plots presenting the effect size on W-scores of the cerebrospinal fluid volume of each region of interest. The plots show the effect sizes for comparisons between CU individuals and FTD subtypes in **(A)**, as well as DAT patients in **(B)**. This highlights the magnitude of differences in the distribution of W-scores across the group comparison. The dots in plot represent Cohen’s *d* values, and the values on both sides represent 95% confidence intervals. Cohen’s *d* values less than 0.2 are considered “very small,” less than 0.5 “small,” less than 0.8 “Medium,” less than 1.2 “large,” and greater than or equal to 1.2 “very large.” CU, cognitively unimpaired; FTD, frontotemporal dementia; bvFTD, behavioral variant frontotemporal dementia; nfvPPA, nonfluent variant primary progressive aphasia; svPPA, semantic variant primary progressive aphasia; DAT, dementia of Alzheimer’s type; CSF, cerebrospinal fluid; L_Anterior_LV, left anterior lateral ventricle; R_Anterior_LV, right anterior lateral ventricle; L_Posterior_LV, left posterior lateral ventricle; R_Posterior_LV, right posterior lateral ventricle; L_Hippocampal, left hippocampal; R_Hippocampal, right hippocampal; L_Frontal, left frontal; R_Frontal, right frontal; L_Temporal, left temporal; R_Temporal, right temporal; L_Parietal, left parietal; R_Parietal, right parietal; L_Occipital, left occipital; R_Occipital, right occipital; Global, sum of all cerebrospinal fluid regions of interest.

Figure 3 illustrates the effect sizes of W-scores across RoIs for three pairwise comparisons: each FTD subtype versus CU individuals (Figure 3A), and each FTD subtype versus DAT patients (Figure 3B). When compared with CU individuals, most RoIs—except the right occipital lobe—exhibited medium or larger effect sizes in W-scores. In comparison with DAT patients, bvFTD exhibited large effect sizes in the bilateral frontal lobes and medium effects in the anterior LVs, right temporal lobe, and right hippocampal region. The nfvPPA subtype demonstrated large effect sizes in the left frontal lobe, with additional medium effects in the right frontal, left parietal lobes, and left anterior LV. In the svPPA group, the most notable effect was observed in the left hippocampal region, followed by the bilateral temporal lobes.

**Figure 4:**
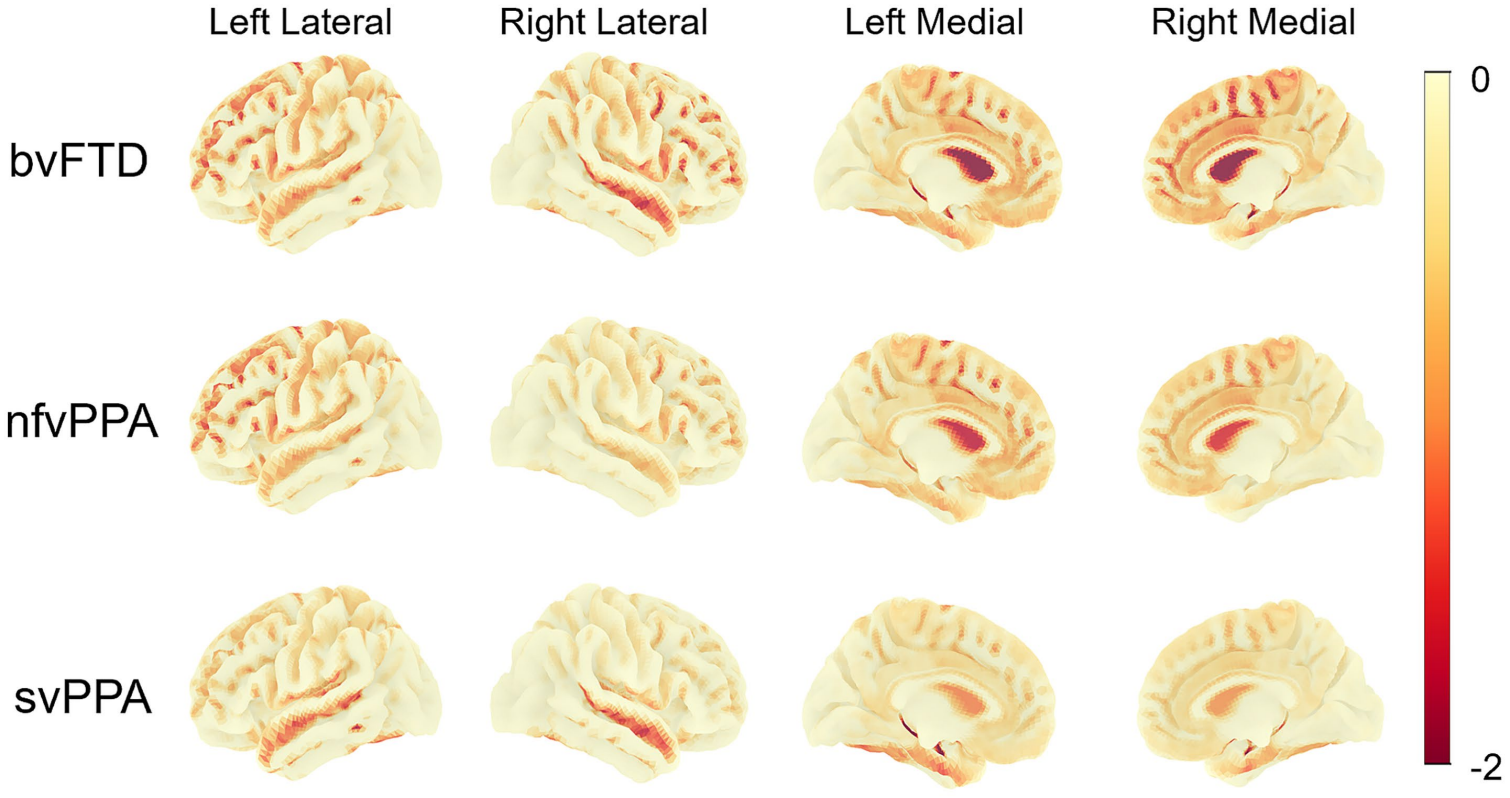
Surface maps visualizing W-score based brain atrophy patterns across FTD subtypes. The surface maps show group-averaged W-scores for each FTD subtype, projected onto a standardized cortical surface, illustrating the spatial distribution of brain atrophy based on CSF volume. FTD, Frontotemporal dementia; bvFTD, behavioral variant frontotemporal dementia; nfvPPA, nonfluent variant primary progressive aphasia; svPPA, semantic variant primary progressive aphasia.

Consistent with these results, surface map visualizations provided intuitive spatial representations of the key regional atrophy patterns in FTD subtypes (Figure 4). In bvFTD, there was widespread bilateral involvement of the frontal lobes, with atrophy extending into the parietal, temporal lobes, as well as anterior LVs. The nfvPPA exhibited prominent atrophy in the left frontal and parietal lobes, along with the anterior LV, aligning with its typical left-hemisphere dominance. In contrast, svPPA was marked by focal atrophy predominantly localized to the temporal lobes.

## Discussion

In this study, regional atrophy patterns among FTD subtypes were investigated using a deep learning-based segmentation model that quantifies CSF volume. The findings revealed significant differences in W-scores among diagnostic groups, demonstrating the algorithm’s effectiveness in capturing region-specific atrophy patterns. These results confirmed the initial hypothesis that each FTD subtype exhibits distinct spatial patterns of brain atrophy. Such precise characterization of atrophy may have clinical utility in improving differential diagnosis and disease monitoring in patients with FTD.

The results revealed clear and distinct atrophy patterns across FTD subtypes. Compared to DAT, bvFTD showed notably greater atrophy prominently in bilateral frontal regions (large effect sizes, Cohen’s d > 1.2), with a slightly stronger involvement observed in the right hemisphere. Medium-sized effects were also present in bilateral parietal and temporal lobes, anterior lateral ventricles, and hippocampal regions. Interestingly, parietal lobe involvement in bvFTD, although traditionally less emphasized, could reflect more advanced disease stages or clinical heterogeneity within this cohort. In contrast, the nfvPPA subtype demonstrated significant atrophy predominantly in the left frontal (Cohen’s d > 1.2) and left parietal regions, consistent with known left hemisphere-dominant language impairments. Similarly, svPPA showed pronounced left-dominant atrophy in temporal lobes and hippocampal regions, with the left hippocampal region exhibiting notably large effect sizes (Cohen’s d > 1.2). Such marked left-sided hippocampal involvement in svPPA aligns closely with previous findings, highlighting medial temporal lobe involvement beyond classic AD pathology. These observations are consistent with previously reported subtype-specific atrophy patterns (Risacher and Saykin, 2013; Ghosh and Lippa, 2015; Eldaief et al., 2023; Taylor et al., 2025), further reinforcing the validity and clinical relevance of the proposed approach (see Supplementary Table 2).

The 3D surface maps also provided compelling visual validation of these subtype-specific patterns, clearly demonstrating left-lateralized atrophy in nfvPPA and svPPA and widespread, anterior-predominant atrophy in bvFTD. These visualizations enhance the interpretability and clinical relevance of the observed structural differences, emphasizing the spatial specificity achievable by this method.

To further ensure the robustness of these findings, an additional propensity score matching (PSM) analysis was conducted using age, sex, and education as matching factors. After excluding cases with missing education data, 331 CU and 331 DAT participants were matched to 342 FTD participants with complete data (Supplementary Table 3). This adjustment reduced group size disparities and allowed for more balanced comparisons. Some regional differences that were previously significant (e.g., left temporal region in bvFTD, right frontal and left parietal regions in nfvPPA, and left frontal region in svPPA) were no longer observed after matching (Supplementary Figure 2) and the overall effect sizes were somewhat reduced (Supplementary Figure 3). However, the spatial patterns of atrophy remained largely consistent with the primary analysis.

These findings extend previous work conducted in Alzheimer’s disease, where stage-specific atrophy patterns—particularly in bilateral temporal lobes, lateral ventricles, and hippocampal regions—were demonstrated across a cognitive continuum from cognitively unimpaired individuals to patients with mild cognitive impairment and DAT. In the current study, quantitative CSF-based measures derived from the algorithm revealed significant hemispheric asymmetries—particularly within the left hemisphere—across FTD subtypes when compared with CU and DAT groups. Importantly, these findings were achieved using a quantification approach distinct from conventional methods (Scheltens et al., 1995; Ashburner and Friston, 2000; Fischl and Dale, 2000; Rosen et al., 2002; Kipps et al., 2007), yet consistently capturing relevant structural disease signatures. The ability to detect such detailed patterns highlights this method’s potential as a robust structural biomarker, particularly valuable for clinical evaluations in cases involving focal atrophy of the frontal and temporal lobes. This ensures reliable, scalable analysis across different clinical settings and patient groups. Given its reliance on standard clinical imaging modalities, this approach could be easily integrated into routine neuroimaging workflows, supporting clinicians in the early and differential diagnosis of dementia subtypes. With further validation, the algorithm may also offer potential benchmarks for future adaptations to more accessible imaging modalities, such as 2D MRI or even CT scans.

A major strength of this study is the practical capability of the deep learning-based method to quantify brain atrophy directly from routine MRI scans without complex preprocessing. However, several limitations should be acknowledged. The analysis relied solely on CSF volume, age, and sex, without incorporating additional factors (Sakka et al., 2011; Zhang et al., 2022) or direct measures of neurodegeneration such as cortical thickness or voxel-based morphometry. To complement the primary analyses, cortical thickness data in a subset of participants were also examined, which revealed broadly consistent and biologically plausible regional patterns with CSF-based measures (see Supplementary Figures 4, 5). Future work may examine the combined use of CSF- and GM-based features to improve both anatomical specificity and clinical interpretability. Additionally, the lack of clinical and cognitive data limits structure–function interpretations, and the cross-sectional design prevents assessment of longitudinal changes. Finally, the single-center design restricts generalizability, highlighting the need for external validation. Nonetheless, our findings demonstrate robust subtype-specific atrophy patterns, underscoring the method’s clinical relevance and value for dementia research.

In summary, this study demonstrated significant differences in regional atrophy patterns across FTD subtypes using a CSF-based deep learning algorithm. By focusing on clinically relevant RoIs, this simple and practical approach facilitates intuitive interpretation, rapid clinical integration, and consistent application across diverse settings. With further validation, this method holds significant potential as a robust structural biomarker for enhancing differential diagnosis and monitoring disease progression.

## Data Availability

The anonymized data for the analyses presented in this report are available from the corresponding author on reasonable request.

## Footnotes

^1^ http://www.r-project.org
