# Supplementary material for: Regional CSF volume quantification using deep learning for comparative analysis of brain atrophy in frontotemporal dementia subtypes
Source: Front Aging Neurosci. 2025 Sep 22;17:1631640. doi: 10.3389/fnagi.2025.1631640 (PMC12497847; doi:10.3389/fnagi.2025.1631640)
Supplement: Supplementary file 1 [file Supplementary_file_1.docx]

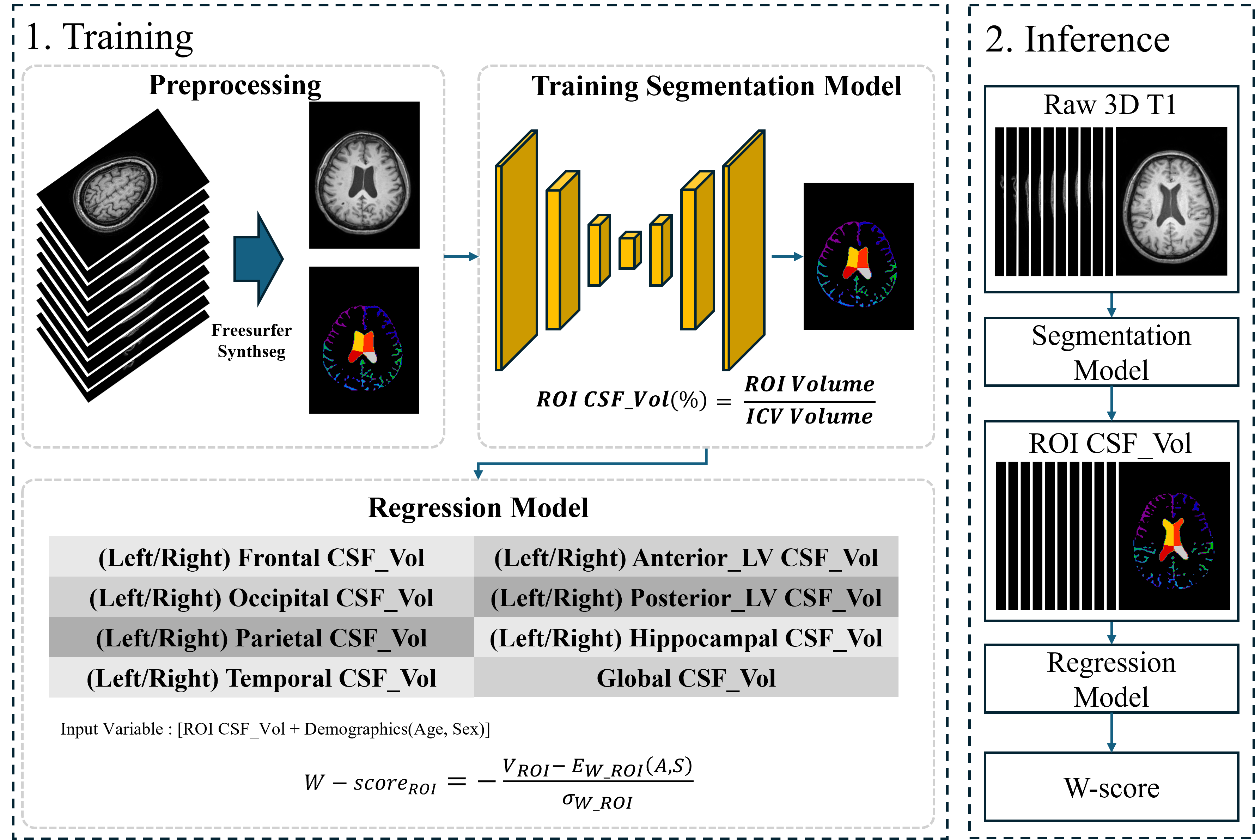
**Supplementary Fig.1. Overview of the algorithm pipeline.** The figure illustrates the algorithmic pipeline for computing CSF RoI volumes and corresponding W-scores. During the training stage, a 3D segmentation model was trained to segment 14 CSF-related RoIs and the ICV from T1-weighted MRI scans. The extracted RoI volumes were then modeled as functions of age and sex using a cognitively unimpaired reference group to establish normative W-score models. In the inference stage, the trained model estimates RoI volumes and ICV from raw 3D T1-weighted images. W-scores are subsequently computed by comparing the observed volumes to the normative models, adjusting for age and sex. Abbreviations: RoI = region of interest; CSF = cerebrospinal fluid; ICV = intracranial volume

Supplementary Table 1. Demographic information of dataset for segmentation model

| **Cohort** | **N** | **Female** | **Age** |
| --- | --- | --- | --- |
| **Samsung Medical Center** | | | |
| SMC | 1,889 | 1,119 | 71.1±9.4 |
| **Other public dataset** | | | |
| ADNI | 250 | 104 | 75.8±6.8 |
| ICBM | 157 | 82 | 41.6±15.1 (-3) |
| IXI | 581 | 313 | 48.4±16.8 (-15) |
| OASIS-3 | 149 | 88 | 75.4±7.5 |
| Total | 3026 | 1706 | 65.9±15.6 |

A total of 3,026 participants were obtained from multiple cohorts, including the Samsung Medical Center, the Alzheimer’s Disease Neuroimaging Initiative, the International Consortium for Brain Mapping, the Information Extraction from Images project, and the Open Access Series of Imaging Studies. Three‑dimensional T1‑weighted magnetic resonance imaging scans were acquired and employed for model training and validation. Missing demographic information for certain datasets is reported in parentheses.

Abbreviations: SMC = Samsung Medical Center; ADNI = Alzheimer’s Disease Neuroimaging Initiative; ICBM = International Consortium for Brain Mapping; IXI = Information extraction from Images; OASIS = Open Access Series of Imaging Studies; N = number of subjects.

Supplementary Table 2. Summary of previously reported brain atrophy patterns in FTD subtypes and their consistency with the current study findings

| Study | Method | Reported Atrophy Patterns | Consistency with current study |
| --- | --- | --- | --- |
| Risacher and Saykin 2013 | Literature review | bvFTD: frontal lobe, anterior cingulate, anterior insula, thalamus  nfvPPA: left inferior frontal, insula, premotor cortex, temporal lobe  svPPA: Asymmetrical atrophy of the anterior, medial & inferior temporal lobes(left > right) | Bilateral frontal involvement in bvFTD aligns with reported frontal lobe atrophy; Left-dominant patterns in nfvPPA (frontal, temporal) and svPPA (temporal predominance) match current findings |
| Ghosh and Lippa 2015 | Literature review | bvFTD: orbital, mesial frontal lobe, anterior temporal lobe  nfvPPA: left frontal, perisylvian atrophy, left basal ganglia  svPPA: temporal lobe atrophy (anteriorly, involving polar, anterior parahippocampal, fusiform region including perirhinal cortex); bilateral but typically asymmetric and severe on left side | bvFTD orbital/mesial frontal atrophy corresponds to bilateral frontal findings; Left frontal patterns in nfvPPA and left-predominant temporal atrophy in svPPA support current hemispheric asymmetry results |
| Eldaief, Brickhouse et al. 2023 | Surface-based cortical thickness analysis | bvFTD: Superior frontal gyrus, dorsomedial prefrontal cortex, dorsal, subgenual anterior cinglate cortex, temporal pole, middle temporal gyrus, anterior medial temporal lobe | bvFTD superior frontal gyrus and dorsomedial prefrontal cortex atrophy directly aligns with bilateral frontal atrophy patterns observed in current study |
| Taylor, Bocchetta et al. 2025 | Subtype and Stage Inference(SuStaIn)-based unsupervised machine learning clustering | nfvPPA: left precentral gyrus, left frontal lobe, parietal lobe, insula cortex, temporal lobe  svPPA: left inferior temporal cortex, left temporal trgions, insula cortex, right temporal regions, frontal lobe | Left frontal/parietal dominance in nfvPPA and left temporal cortex involvement in svPPA precisely match current study's left-hemisphere dominant pattern |
| Lu, Mendez et al. 2013 | tensor-based morphometry | bvFTD: superior and middle frontal lobes, anterior temporal lobes, posterior regions  nfvPPA: frontal, temporal, parietal regions  svPPA: anterior, middle, inferior temporal lobes, anterior, posterior lateral ventricles, frontal regions | bvFTD bilateral frontal involvement matches current bilateral frontal atrophy; nfvPPA frontal and parietal patterns align with left frontal/parietal findings; svPPA temporal lobe predominance corresponds to left temporal/hippocampal atrophy observed in current study |
| Planche, Mansencal et al. 2023 | Longitudinal trajectory modeling | bvFTD: bilateral amygdala, striatum, anterior insula, hippocampus, thalamus, right temporal pole, middle temporal gyrus, bilateral prefrontal cortex, orbital gyri, frontal poles  nfvPPA: left striatum, bilateral anterior insula, left thalamus, left operculum, left precuneus, cuneus  svPPA: bilateral amygdala, left temporal pole, hippocampus, anterior insula, middle temporal gyrus, striatum, other left temporal structures, right-lateralization of temporal atrophy | bvFTD bilateral prefrontal cortex and temporal involvement supports current bilateral frontal findings; nfvPPA left-sided striatum, insula, and operculum atrophy aligns with left-dominant patterns; svPPA bilateral amygdala and left temporal structures match current left temporal/hippocampal results; Demonstrates that current cross-sectional findings reflect established longitudinal progression patterns |

This table summarizes the main findings from selected prior studies investigating regional brain atrophy patterns in behavioral variant frontotemporal dementia (bvFTD), nonfluent variant primary progressive aphasia (nfvPPA), and semantic variant primary progressive aphasia (svPPA). Abbreviations: bvFTD = behavioral variant frontotemporal dementia; nfvPPA = nonfluent variant primary progressive aphasia; svPPA = semantic variant primary progressive aphasia.

Supplementary Table 3. Characteristics of participants after propensity score matching

| Characteristics | CU  (N=331) | bvFTD  (N=137) | nfvPPA  (N=70) | svPPA  (N=135) | DAT  (N=667) |
| --- | --- | --- | --- | --- | --- |
|  |  |  |  |  |  |
| Age, mean±SD, years^1^ | 68.0±7.5 | 66.6±10.7 | 68.8±8.6 | 67.0±8.6 | 68.5 ±7.5 |
| Sex, female, N(%)^2^ | 153  (46.2%) | 58  (42.3%) | 37  (52.9%) | 66  (48.9%) | 172  (52.0%) |
| Years of education, mean±SD^1^ | 11.6±4.4 | 11.4±4.5  (N=134‡) | 11.5±4.3  (N=66‡) | 11.4±4.4  (N=131‡) | 11.3±4.5 |
| MMSE, mean±SD^1^ | 28.3±1.5 | 20.5±6.1^*^  (N=108‡) | 20.6±7.0^*^  (N=53‡) | 19.1±8.8^*^  (N=113‡) | 19.1±5.4 |
| ICV, mean±SD, mL^1^ | 1,520.4  ±135.3 | 1,505.0  ±139.9 | 1,500.6  ±108.0 | 1,482.8  ±129.1^*^ | 1,478.6  ±136.5 |
| Values are expressed as mean ± standard deviation (SD) or number (%) as appropriate. Statistical comparisons for continuous variables^1^ were performed using analysis of variance (ANOVA) followed by Bonferroni post hoc tests. Categorical variables^2^ were compared using Pearson’s Chi-squared test. A p-value < 0.05 was considered significant.  ‡ Sample sizes for Years of education and MMSE variables differ from the No. of participants due to missing data:  - Years of education: bvFTD (n=134), nfvPPA (n=66), svPPA (n=131).  - MMSE: bvFTD (n=108), nfvPPA (n=53), svPPA (n=113).  Summary statistics for the overall cohort are provided in the text.  Abbreviations: CU, cognitively unimpaired; bvFTD, behavioral variant frontotemporal dementia; nfvPPA, nonfluent variant primary progressive aphasia; svPPA, semantic variant primary progressive aphasia; DAT, dementia of Alzheimer’s type; N, number of subjects; SD, Standard deviation; MMSE, Mini–Mental State Examination; ICV, intracranial volume, Asterisk (*), significant difference with cognitively unimpaired individuals; Dagger (†), significant difference with dementia of Alzheimer’s type patients | | | | | |

**
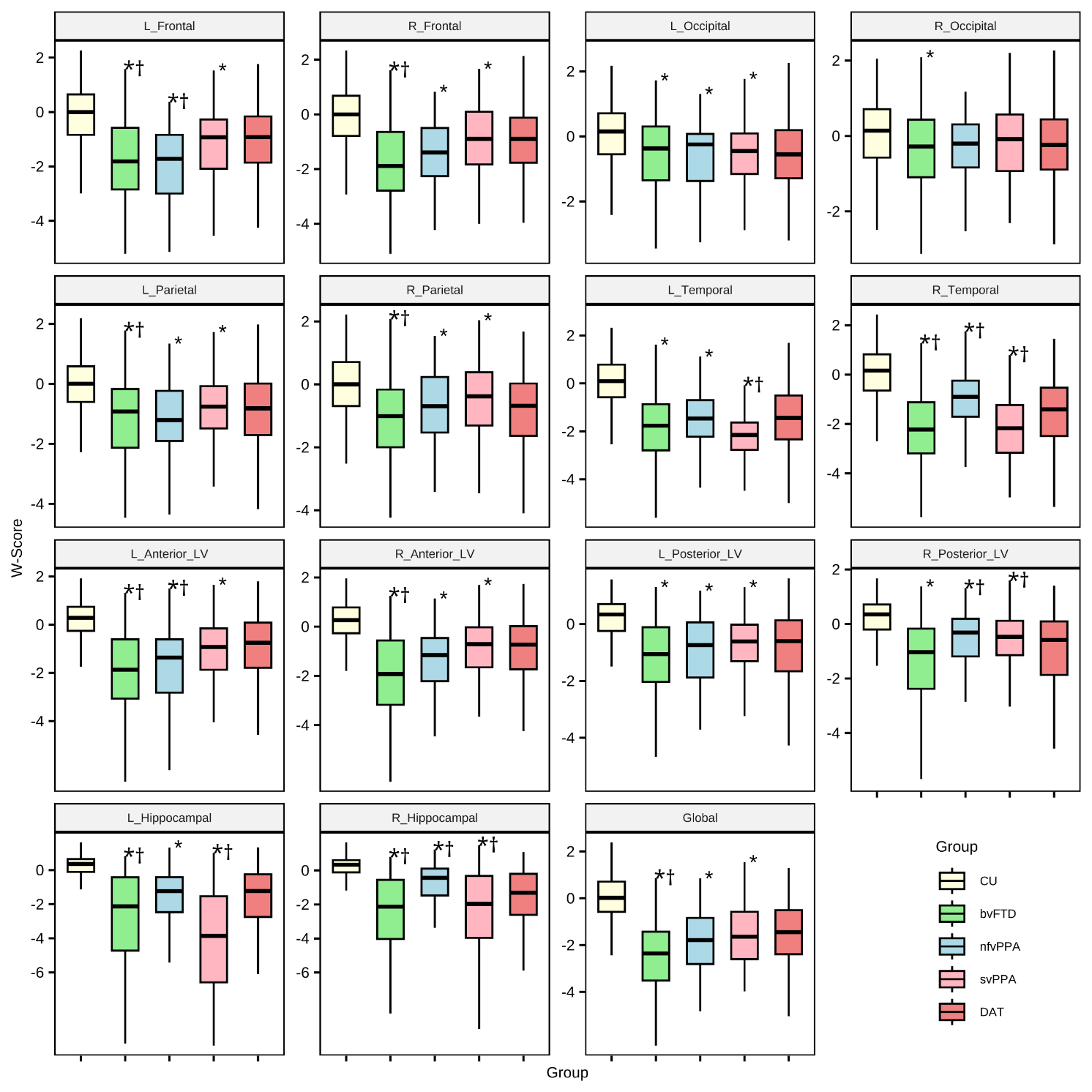
Supplementary Fig.2. Boxplots of W-scores for cerebrospinal fluid volume of each region of interest after propensity score matching.** An asterisk (*) indicates significant difference with CU individuals and dagger (†) indicates significant difference with DAT patients. For visual convenience, W-scores beyond ±3 standard deviations were excluded from the boxplots, without affecting the underlying statistical analysis. Abbreviations: CU, cognitively unimpaired; bvFTD, behavioral variant frontotemporal dementia; nfvPPA, nonfluent variant primary progressive aphasia; svPPA, semantic variant primary progressive aphasia; DAT, dementia of Alzheimer’s type; CSF, cerebrospinal fluid; L_Anterior_LV, left anterior lateral ventricle; R_Anterior_LV, right anterior lateral ventricle; L_Posterior_LV, left posterior lateral ventricle; R_Posterior_LV, right posterior lateral ventricle; L_Hippocampal, left hippocampal; R_Hippocampal, right hippocampal; L_Frontal, left frontal; R_Frontal, right frontal; L_Temporal, left temporal; R_Temporal, right temporal; L_Parietal, left parietal; R_Parietal, right parietal; L_Occipital, left occipital; R_Occipital, right occipital; Global, sum of all cerebrospinal fluid regions of interest.


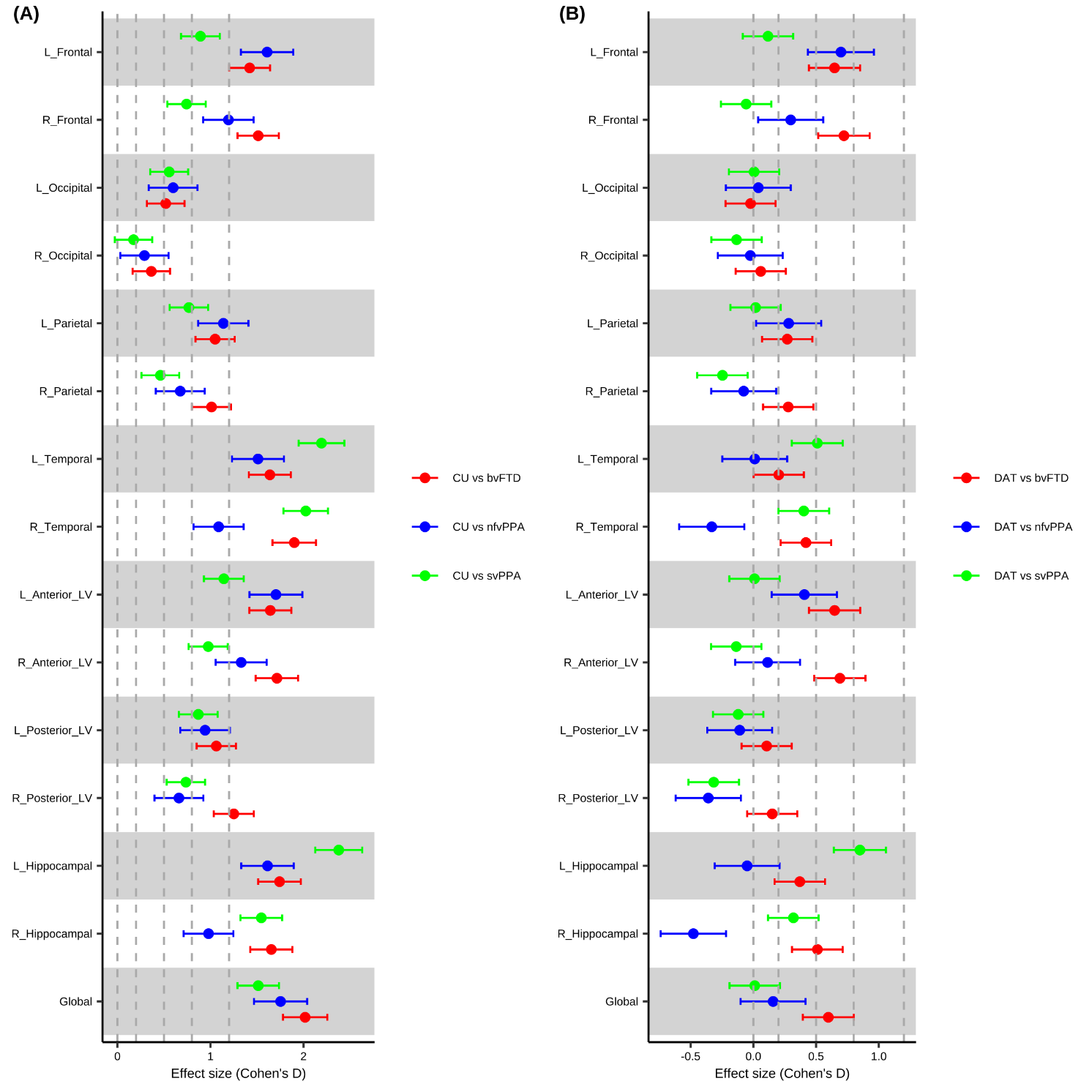


**Supplementary Fig. 3. Forest plots presenting the effect size on W-scores of the cerebrospinal fluid volume of each region of interest after propensity score matching.** An asterisk (*) indicates significant difference with CU individuals and dagger (†) indicates significant difference with DAT patients. For visual convenience, W-scores beyond ±3 standard deviations were excluded from the boxplots, without affecting the underlying statistical analysis. Abbreviations: CU, cognitively unimpaired; bvFTD, behavioral variant frontotemporal dementia; nfvPPA, nonfluent variant primary progressive aphasia; svPPA, semantic variant primary progressive aphasia; DAT, dementia of Alzheimer’s type; CSF, cerebrospinal fluid; L_Anterior_LV, left anterior lateral ventricle; R_Anterior_LV, right anterior lateral ventricle; L_Posterior_LV, left posterior lateral ventricle; R_Posterior_LV, right posterior lateral ventricle; L_Hippocampal, left hippocampal; R_Hippocampal, right hippocampal; L_Frontal, left frontal; R_Frontal, right frontal; L_Temporal, left temporal; R_Temporal, right temporal; L_Parietal, left parietal; R_Parietal, right parietal; L_Occipital, left occipital; R_Occipital, right occipital; Global, sum of all cerebrospinal fluid regions of interest.


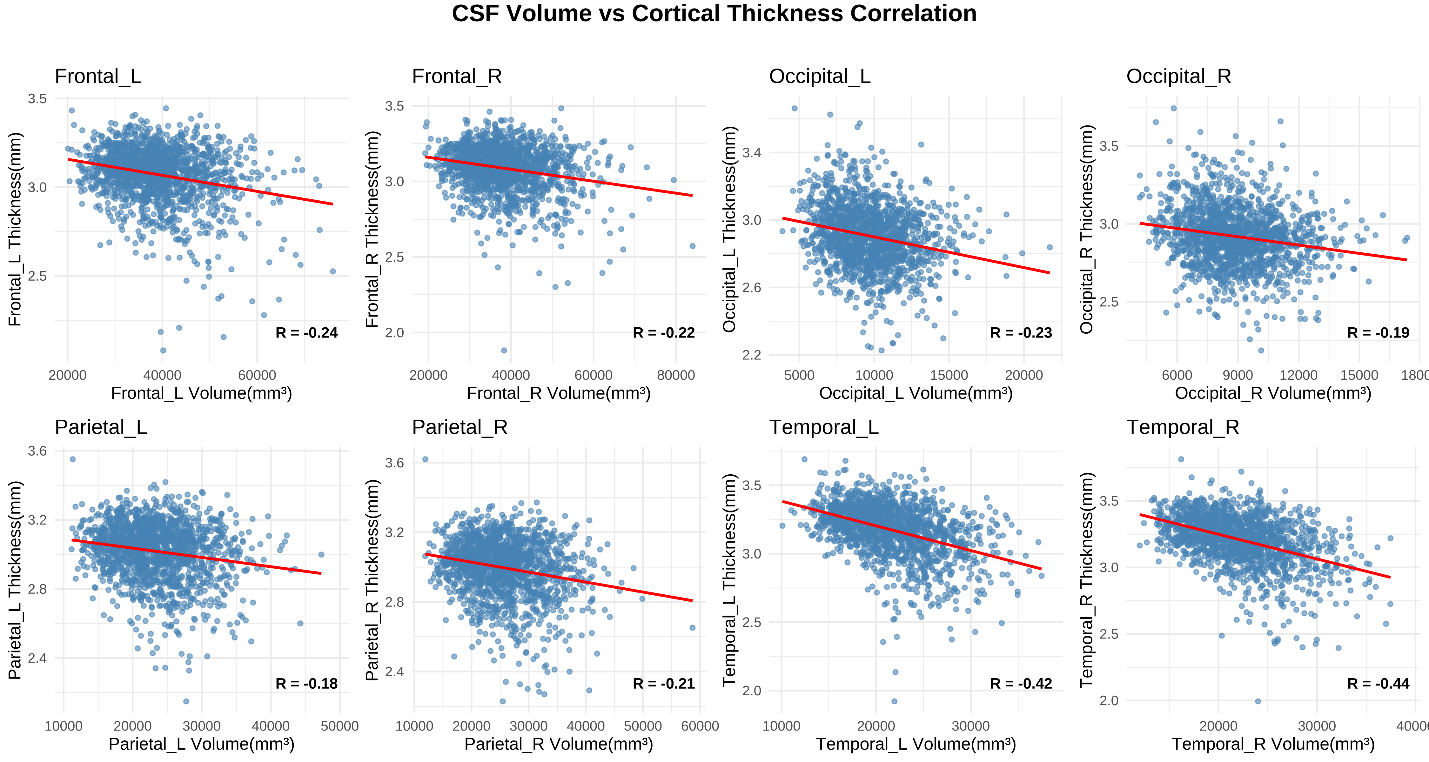
**Supplementary Fig.4. Correlation of CSF volume and cortical thickness across regions of interest.** Scatter plots exhibit correlations for cerebrospinal fluid and cortical thickness. Abbreviations: CSF, cerebrospinal fluid; L_Frontal, left frontal; R_Frontal, right frontal; L_Occipital, left occipital; R_Occipital, right occipital L_Occipital, left occipital; R_Occipital, right occipital; L_Parietal, left parietal; R_Parietal, right parietal L_Temporal, left temporal; R_Temporal, right temporal.

**
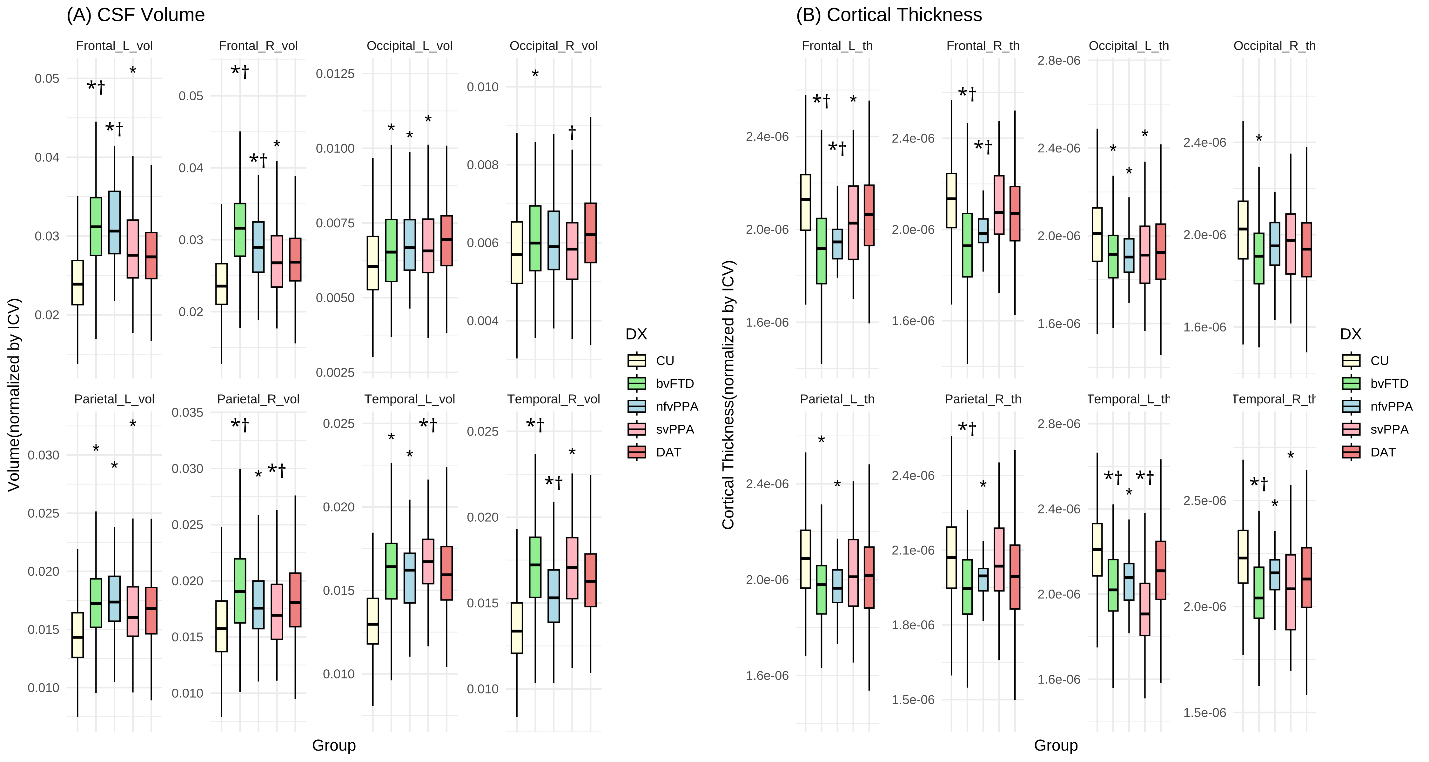
Supplementary Fig.5. Boxplots of cerebrospinal fluid volume and cortical thickness of each region of interest.** An asterisk (*) indicates significant difference with CU individuals and dagger (†) indicates significant difference with DAT patients. For visual convenience, W-scores beyond ±3 standard deviations were excluded from the boxplots, without affecting the underlying statistical analysis. Abbreviations: CU, cognitively unimpaired; bvFTD, behavioral variant frontotemporal dementia; nfvPPA, nonfluent variant primary progressive aphasia; svPPA, semantic variant primary progressive aphasia; DAT, dementia of Alzheimer’s type; CSF, cerebrospinal fluid; L_Frontal, left frontal; R_Frontal, right frontal; L_Occipital, left occipital; R_Occipital, right occipital L_Occipital, left occipital; R_Occipital, right occipital; L_Parietal, left parietal; R_Parietal, right parietal L_Temporal, left temporal; R_Temporal, right temporal.
